# Outbreaks and Surveys: A Dilogy

**DOI:** 10.3201/eid2307.170315

**Published:** 2017-07

**Authors:** Jay S. Keystone

**Affiliations:** Toronto General Hospital–Medicine, Toronto, Ontario, Canada

**Keywords:** infectious disease outbreaks, infectious disease prevalence, infectious disease epidemiology, outbreaks, surveys

For medical academics and health workers, the task of efficiently gathering published data concerning outbreaks and epidemics can be overwhelming and time-consuming, always at the risk of excluding crucial information. As of February 2017, a single source has compiled a staggering amount of medical information into a comprehensive collation of infectious disease outbreaks and surveys. 

GIDEON Guide to Outbreaks (https://www.gideononline.com/ebooks/outbreaks) was “written” by a computer program that converts database entries (https://www.gideononline.com) into e-book format. The guide describes 21,365 pandemics, epidemics, and case-clusters summarized in 5,120 tables, each devoted to a specific disease–country grouping (e.g., Salmonellosis–Albania …Salmonellosis–Zimbabwe). Charts detail outbreak year, region (e.g., city, province), affected population, setting, number of cases, number of deaths, pathogen, syndrome, vehicle/source, and electronically linked references. Where relevant, chapters begin with a listing of global and regional pandemics or unusually extensive or iconic events (i.e., the Typhoid Mary outbreaks, the Black Death). To facilitate searching within the book, introductory charts summarize total outbreaks, earliest recorded outbreaks, links to specific countries, and indexing terms (including foreign language disease designations).

The format of GIDEON Guide to Surveys (https://www.gideononline.com/ebooks/surveys) is similar to that of the Outbreaks book; it chronicles all published studies of disease prevalence (e.g., hookworms in Tanzania, gonorrhea in the United States) and seroprevalence (HIV in India, toxoplasmosis in Brazil). The current edition includes 53,917 studies. 

Charts in Outbreaks group events by year, and charts in Surveys list entries by demographic group (e.g., blood donors, children, STD patients). Sorting these tables by user-selected parameters (region, case numbers) is not possible in the e-book format, but the option is available in the online version of the program.

Both books are updated yearly. Both incorporate data for zoonotic diseases among animals, but not to the extent allotted to human illness. The list of diseases and their references (many from PubMed) included in these charts is exhaustive and includes such illnesses and conditions as Spondweni virus infection, coenurosis, philophthalmosis, and Wesselsbron disease. On the other hand, these books do not address healthcare-associated infections or infections caused by a growing list of bacteria of >1,600 taxa associated with human disease (e.g., *Acinetobacter*, *Klebsiella*, enterococci). The most useful features of these 2 e-books are their exhaustive and up-to-date content, intuitive organization, and encyclopedic format. 

It would be helpful if relatively major events were displayed in bold text. For example, Outbreaks lists 2 outbreaks of trichinosis that occurred in France in 1985, one of 21 and the other of 1,073 cases, without special visual treatment or comment. Those of us accustomed to standard books written in lines of text will find these sources difficult to “read.” 

Berger notes that GIDEON Guide to Outbreaks and GIDEON Guide to Surveys are written for use by workers, educators, and students in the fields of infectious diseases and geographic medicine. I am certain that both will prove useful to readers of Emerging Infectious Diseases.

